# Portable light transmission measuring system for preserved corneas

**DOI:** 10.1186/1475-925X-4-70

**Published:** 2005-12-22

**Authors:** Liliane Ventura, Gabriel Torres de Jesus, Gunter Camilo Dablas de Oliveira, Sidney JF Sousa

**Affiliations:** 1 Universidade de São Paulo, Laboratório de Instrumentação Oftálmica, Departamento de Engenharia Elétrica – EESC-USP, São Carlos, SP, Brasil; 2 Universidade de São Paulo, Laboratório de Física Oftálmica, Departamento de Oftalmologia – FMRP/USP, Ribeirão Preto, SP, Brasil

## Abstract

**Background:**

The authors have developed a small portable device for the objective measurement of the transparency of corneas stored in preservative medium, for use by eye banks in evaluation prior to transplantation.

**Methods:**

The optical system consists of a white light, lenses, and pinholes that collimate the white light beams and illuminate the cornea in its preservative medium, and an optical filter (400–700 nm) that selects the range of the wavelength of interest. A sensor detects the light that passes through the cornea, and the average corneal transparency is displayed. In order to obtain only the tissue transparency, an electronic circuit was built to detect a baseline input of the preservative medium prior to the measurement of corneal transparency. The operation of the system involves three steps: adjusting the "0 %" transmittance of the instrument, determining the "100 %" transmittance of the system, and finally measuring the transparency of the preserved cornea inside the storage medium.

**Results:**

Fifty selected corneas were evaluated. Each cornea was submitted to three evaluation methods: subjective classification of transparency through a slit lamp, quantification of the transmittance of light using a corneal spectrophotometer previously developed, and measurement of transparency with the portable device.

**Conclusion:**

By comparing the three methods and using the expertise of eye bank trained personnel, a table for quantifying corneal transparency with the new device has been developed. The correlation factor between the corneal spectrophotometer and the new device is 0,99813, leading to a system that is able to standardize transparency measurements of preserved corneas, which is currently done subjectively.

## Introduction

The cornea consists of three layers [1,2]: the epithelium, the stroma, and the endothelium. The epithelium acts as a mechanical protection. The stroma is responsible for 95 % of its thickness, and is composed of bundles of collagen fibers arranged in crossed layers. The regularity of the distribution of collagen fibers, the absence of blood vessels, and the low hydration of the tissue are responsible for its high transparency. Finally, the endothelium is the internal lining of the cornea, consisting of a single cell layer with no capacity for regeneration. It is particularly important in the active preservation of corneal dehydration.

Scars, vascularization, and edema alter corneal transparency. When the vision is decreased significantly by one of these conditions, a corneal transplantation is generally indicated. The choice of the corneal tissue to be used considers the medical history and the blood analysis of the donor to avoid transmittable diseases, and also the morphological features of the three layers of the cornea to exclude any abnormality of the donated tissue. One of the obvious characteristics considered in the examination of the cornea is its overall transparency, which is usually subjectively analyzed with a slit lamp [3]. In order to obtain an objective measurement of this parameter, we present here a device that provides the percentage of light transmitted by the cornea stored in preservative medium.

## Methods

The optical system for measuring the transparency of the preserved cornea is a relatively simple set up. It provides the percentage of total corneal transparency, but does not determine which corneal layer is responsible for the loss of transparency. The system is very effective in objectively measuring the average transparency of the overall cornea.

**Figure 1:**
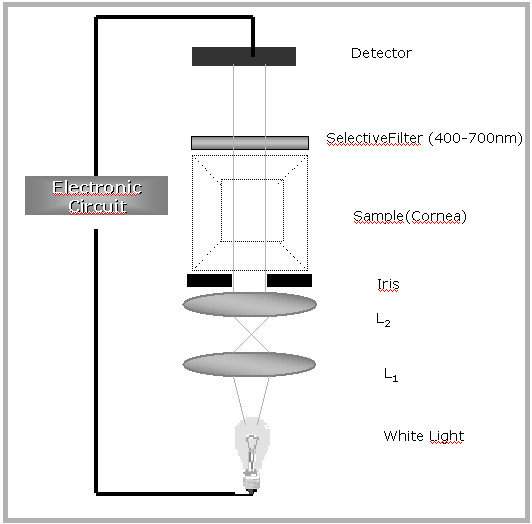
The schematic optics of the system.

**Figure 2:**
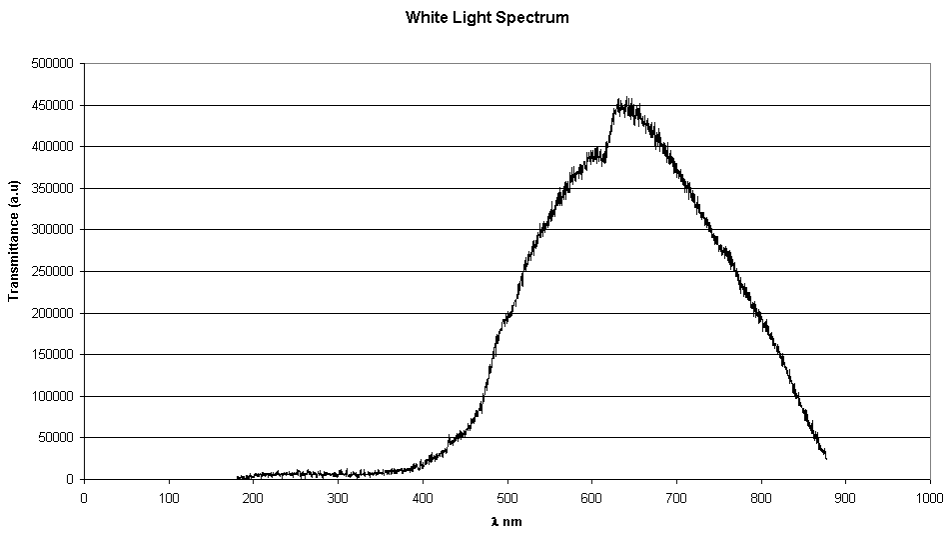
The spectrum of the white light used in the prototype.

The optical system consists of a white light source (15 W, 6 V retinoscope lamp) that passes through a light collimating optical system comprised of two BK7 converging lenses (D = 25.4 mm, F = 50.0 mm, and F = 60.0 mm) and a 3.0 mm pin hole to limit the light passing through the central portion of the cornea (corneal button), a slot for holding the cornea, a filter (D = 25.4 mm, F = 45.0 mm, Visible Achromat, 400–700 nm), and a photodiode detector. Figure 1 shows a schematic illustration the system and the light ray trace. Figure 2 shows the spectrum of the white light used in this system, which is part of our input data, and is equivalent to the spectrum used in the spectrophotometer that the measurements are compared to for evaluation.

The light passes through the cornea stored in preservative medium and the sensor detects the transmitted light. The OPT101 is a monolithic photodiode with on-chip transimpedance amplifier. Output voltage increases linearly with light intensity. The integrated combination of photodiode and transimpedance amplifier on a single chip eliminates the problems commonly encountered in discrete designs, such as leakage current errors, noise pick-up, and gain peaking due to stray capacitance. The 0.23 × 0.23 mm photodiode is operated in the photoconductive mode for excellent linearity and low dark current.

The system is calibrated by adjusting an external knob such that 0 % transmittance corresponds to the absence of light and 100 % corresponds to transmittance through the preservative medium without the cornea.

The variation of the light intensity that reaches the detector induces a change in current. This signal is sent to an analog-to-digital converter and then transmitted to a 3-1/2-digit display, which shows the percentage of light reaching the detector. For calibration of the system, the electronic circuit stores the 0 % and 100 % transmittances of the system determined as described above.

**Figure 3:**
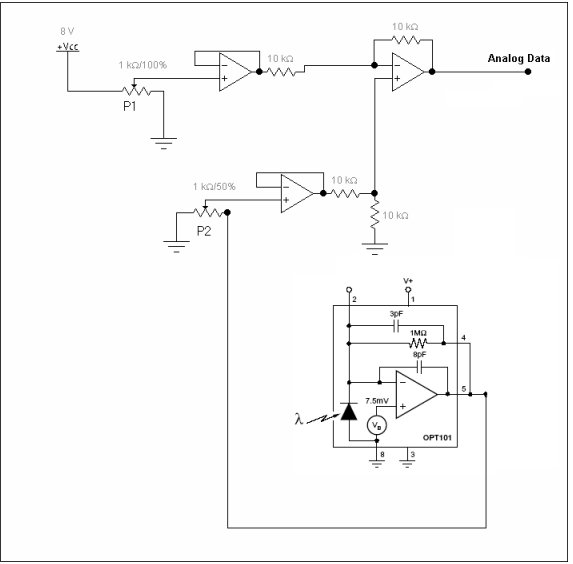
Schematic diagram of the electronic circuit connected to the OPT101 sensor.

The electronics of the prototype can be separated into three basic blocks:

1. *Power supply*: Consists of a 1 A transformer (primary 110/220 V and secondary -12/+12 V). The positive output voltages supply the integrated circuits of the operational amplifier, the A/D circuit, and the OPT101 photodiode; the negative input voltages supply the A/D circuit and the operational amplifiers. The positive output is also a supply for the "7805" integrated circuit, which provides 5 V (stabilized) for obtaining constant brightness of the light.

2. *Control and adjusts*: This block is responsible for manipulating the OPT101 voltage output signal and transmitting the processed signal to the A/D converter in the visualization block. The electronic circuit uses three low-noise HITACHI HA17741 operational amplifiers, configured as two buffers and one comparator. Figure 3 illustrates the electronic circuit connected to the OPT101 sensor.

The two buffers shown in the circuit provide stabilized power to protect both OPT101 and zero reference signals from load effects. The other operational amplifier is configured as a comparator, operating as:

VO = V_OPT _- V_ZREF _    (1)

Where V_OPT _is the OPT101 buffer output signal and V_ZREF _is the zero reference buffer output signal. VO is the block output signal, connected to the A/D converter.

The P1 potentiometer provides adjustment of the zero percent reference level, and the P2 potentiometer adjusts the OPT101 buffer output signal level. The analog output signal is connected to the A/D converter.

Potentiometer P2 is externally available to the user to calibrate the displayed 100 % signal level with the reference preservative medium inserted in the optical system.

For calibration of the system, the electronic circuit stores the 0 % and 100 % transmittances, determined as described above.

3. *Visualization*: To show the percentage of light transmitted, the control block output signal is digitalized and shown as a numeric in a liquid crystal display (LCD). One integrated circuit board provides this using a monolithic A/D converter (ICL 7106) connected to a 3-1/2 digits numeric LCD. The A/D converter has very high input impedance and requires no external display drive circuitry. It has a dual-slope conversion technique that automatically rejects interference signals. It has true differential input and reference zero integrator phase, eliminating over-range hangover and hysteresis effects. High accuracy is obtained by reducing rollover error to less than one count, and zero reading drift to less than 1 μV/°C.

**Figure 4:**
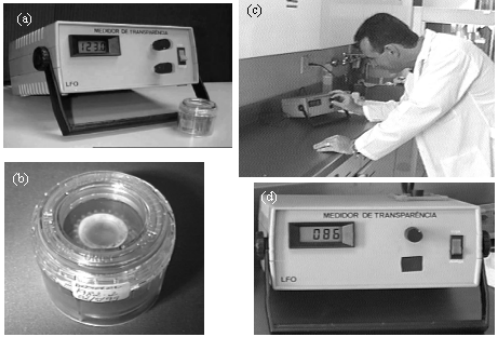
(a) Calibrated prototype just before the measurements of corneal transmittance; (b) The corneoscleral ring in the storage chamber to be placed in the system after adjusting the 100 % baseline; (c) Technician operating the system; (d) Result of the transmission obtained for the examined cornea.

Operating the system is quite simple. The procedure starts by covering the light detector and setting the 0 % transparency of the instrument. Next, the transparency of the vial with the preservative is measured with no cornea inside. This step is used to set the 100 % transparency of the system. Finally, the process is repeated with the cornea inside the preservative solution. The amount of light that reaches the detector is displayed as the percentage of transparency of the cornea. Figure 4a shows the instrument without the storage chamber, just after its adjustment to the nominal 100 % transmittance. Because the stored chamber absorbs 23 % of the incident light, its removal from the system causes the display to show a transmittance of 123 %. Figure 4b shows the preserved cornea to be placed in the system after adjusting the 100 % baseline. Figure 4c shows a technician operating the system, and figure 4d shows the result of the transmission obtained for the examined cornea.

## Results

Fifty human corneas were measured with the new instrument and the results were compared with the corresponding data obtained with conventional spectrophotometry using a dedicated non-commercial spectrophotometer for preserved corneas, as previously developed by Ventura, et al. [5].

**Figure 5:**
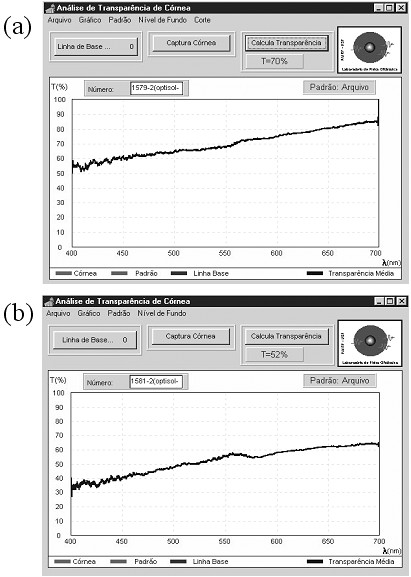
Typical transmission spectra of corneas *in vitro*: (a) a cornea with a relative high transparency of 70 %; (b) a cornea with a relative low transparency of 52 %.

The system for measuring the spectrum of corneal transmission consists of a conventional single beam optical spectroscope fitted with a linear CCD array (2048 sensors) as a detector. The range of measurement is 400 – 700 nm, which is the spectrum of visible light that is to be considered for this type of measurement. A software program acquires the data and provides a graphic interface for the determination of corneal transparency. The resolution is of 9 nm. Typical *in vitro *corneal transmission spectra are shown in Figure 5. Figure 5a shows a cornea with 70 % transparency and Figure 5b a cornea with 52 % transparency. The corneal transparency is obtained by integrating the area under the spectra profile, which is automatically done by the system software. The spectrum profiles are similar, but the intensities and therefore the areas under the graphic signals are quite different.

In order to determine the system accuracy, comparison measurements were made with the prototype and the spectrophotometer. The results were compared for the 400–700 nm range.

**Figure 6:**
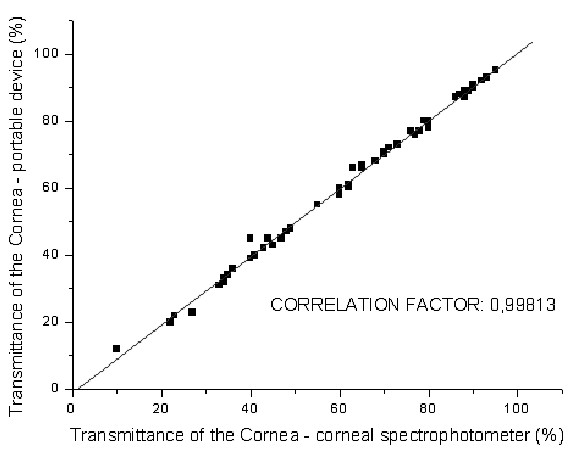
Graphic correlation of the measurements of corneal transmittance of white light obtained with a spectrophotometer and with a new instrument for assessing corneal transparency. Fifty corneas were used in this experiment.

Figure 6 presents the graphic correlation of the measurements of the corneal transmittance of white light obtained with a spectrophotometer and with the new instrument for assessing corneal transparency.

The correlation factor between the corneal spectrophotometer and the new device is 0,99813.

## Discussion

Some back-reflection and light scattering by the cornea are present. However, they do not influence the measurements, since they do not propagate towards the sensor. The important portion of the light to be detected for donated corneas is exclusively the transmittance.

As can be easily seen on figure 6, the coincidence of measurements is quite impressive. This result suggests that the new instrument gives reliable information about corneal transparency with a simpler and cheaper method. Thus, the cornea does not need to undergo a spectroscopy procedure, and the examinations is completed within short time.

The percent corneal transparency obtained with the new instrument was also compared with the subjective classification of corneal transparency as done by the trained personnel from the eye bank that provided the tissue. The results obtained with those same fifty corneas of the first experiment are shown in Table 1. These results are important because they lend clinical meaning to the numbers obtained with the new instrument.

**Table 1:** Corneal transparency objectively assessed with a new instrument for measuring corneal transparency and the corresponding subjective classification done by trained personnel. Corneal transparency objectively assessed with a new instrument for measuring corneal transparency and the corresponding subjective classification done by trained personnel.

Instrument's measurement	Subjective classification
> 75 %	Excellent
45 % – 60 %	Reasonable
< 45 %	Poor

Another benefit of the new measurement system is that it permits accurate transparency measurements by for non-experienced personnel at the eye bank, leading to standard measurements of corneal transparency.

The portability (20 cm × 25 cm × 8 cm) of the system is important in encouraging eye banks to performing objective transmittance measurements.

## Conclusion

The system is quite easy to manipulate and provides objective information on corneal transparency. The correlation factor between the corneal spectrophotometer and the new device demonstrates its accuracy. This means that this portable device is accurate enough to substitute all the optics, CCD arrays, electronics, and optical alignments involved in spectrophotometry used for this kind of measurement.

It should be clear that a spectrophotometer dedicated for corneal measurements is not commercially available. However, measurements in regular spectrophotometers may be performed, although more operator expertise is needed, and they are not as cheap nor as simple as the prototype here presented.

This system not only improves the criteria for the selection of corneas to be used in transplantations, but also raises the opportunity for further studies. For example, it allows the study of the influence on the transparency of the donor corneas of factors such as age, pigment deposits, and endothelial counting. However, its primary application is in eye banks to standardize the evaluation of corneal transparency that currently is done subjectively.

## Authors' contributions

Liliane Ventura is the author of the idea and making the optical parts and the algorithm for the electronics to be implemented, Gabriel Torres de Jesus and Gunter C.D. Oliveira are responsible for implementing the electronics, and Sidney J. Faria e Sousa is the medical doctor that has evaluated the corneas in the prototype, spectrophotometer and subjectively.
